# Seizure adequacy and safety of remimazolam in electroconvulsive therapy for patients with psychiatric disorders: a double-blind randomized crossover trial

**DOI:** 10.1186/s12888-026-08202-x

**Published:** 2026-05-22

**Authors:** Tianqi Shen, Xingxing Yin, Yongming Zhang, Feng Wang, Haipeng Wang, Xiufang Shi, Yanmiao Qiu, Jia Li, Xinhong Du

**Affiliations:** 1 Department of Anesthesiology, No. 984 Hospital of the PLA, Beijing, China; 2 Combat Trauma Center, No. 984 Hospital of the PLA, Beijing, China; 3 Psychiatry and Psychology Center, No. 984 Hospital of the PLA, Beijing, China; 4 Department of Psychiatry, No. 984 Hospital of the PLA, Beijing, China; 5 Psychiatric Intensive Care Unit, No. 984 Hospital of the PLA, Beijing, China

**Keywords:** Electroconvulsive therapy, Remimazolam, Propofol, Psychiatric disorders

## Abstract

**Introduction:**

Electroconvulsive therapy (ECT) is an established and effective treatment for patients with medication-resistant psychiatric disorders. However, the optimal choice of anesthetic agent for ECT induction remains a subject of ongoing debate. Remimazolam, a novel ultra-short-acting benzodiazepine, has yet to be systematically evaluated in the context of ECT anesthesia.

**Method:**

We conducted a prospective, double-blind, randomized, crossover trial in hospitalized patients scheduled for ECT. Participants were randomly assigned to different treatment sequences, receiving either remimazolam (0.2 mg/kg) or propofol (2 mg/kg) for anesthesia induction in a crossover manner. The primary outcome was EEG seizure duration. Secondary outcomes included seizure adequacy endpoint, anesthesia-related parameters and safety profiles. All analyses were performed using generalized linear mixed-effects models, with treatment condition, stimulus dose, and treatment sequence as fixed effects and participant-specific random intercepts.

**Results:**

A total of 284 ECT sessions from 56 patients were included in the analysis. The remimazolam group demonstrated significantly longer EEG seizure duration (37.0 s vs. 18.5 s; *p* < 0.001) and EMG seizure duration (15.0 s vs. 9.0 s, *p* < 0.001), higher average seizure energy index (24,767.4 µV^2^ vs. 15,702.7 µV^2^; *p* < 0.001), and greater maximum sustained power (38,197.7 µV^2^ vs. 30,833.7 µV^2^; *p* < 0.001). However, remimazolam was associated with a higher incidence of post-ictal hypertension and tachycardia, as well as prolonged induction and recovery time.

**Conclusion:**

Remimazolam is an effective anesthetic induction agent for ECT, offering superior seizure quality metrics compared to propofol. However, clinicians should remain vigilant regarding the potential for post-ictal cardiovascular hyperdynamic states.

**Trial registration:**

Chinese Clinical Trial Registry (number: ChiCTR2500114332; date: December 10, 2025).

**Supplementary Information:**

The online version contains supplementary material available at 10.1186/s12888-026-08202-x.

## Introduction

Mental disorders represent a leading cause of global disease burden [1, 2]. Electroconvulsive therapy (ECT) is a well-established medical intervention with a long history in the treatment of diverse psychiatric conditions [3], and has been validated as an effective treatment for severe major depression, mania, catatonia, and clozapine-resistant schizophrenia [4, 5]. The procedure is performed under general anesthesia, during which cerebral electrical stimulation induces a therapeutic seizure, while anesthetic agents ensure adequate anesthesia and neuromuscular blockade [6]. Notably, multiple anesthetic factors—including the choice of sedative-hypnotic agents—can significantly modulate seizure quality [7, 8].

Propofol is a widely used intravenous anesthetic for ECT in China [9–11]. Owing to its favorable induction and sedative-hypnotic properties, which enable rapid and smooth induction of anesthesia, it has become one of the most commonly administered intravenous agents for ECT [12–15]. However, the anticonvulsant properties of propofol may elevate seizure threshold, compromise seizure duration [15] and may cause pain at the injection site [16]. Ketamine demonstrates rapid antidepressant effects [17] and prolongs seizure duration [12, 18] but can reduce hemodynamic stability and increase intracranial pressure [19] as well as prolonged recovery times [20, 21]. Etomidate represents another sedative-hypnotic agent capable of extending seizure duration [22]. However, due to its adrenocortical suppression and immunosuppressive effects [23, 24], cumulative effects may occur with the repeated dosing regimen required for ECT, potentially being more pronounced in vulnerable populations.

Remimazolam, a ultra-short-acting benzodiazepine [25], has gained widespread adoption for procedural sedation in gastrointestinal endoscopy [16] and bronchoscopy [26], as well as for induction and maintenance of general anesthesia, owing to its rapid hydrolysis by tissue esterases [27] and favorable hemodynamic and respiratory profiles [28]. It offers an anesthetic regimen devoid of adrenocortical suppression and amenable to rapid reversal with flumazenil, while maintaining hemodynamic stability comparable to etomidate [29]. Compared with propofol, its shorter context-sensitive half-time, lower incidence of injection pain, and organ-independent metabolism may offer advantages in patients with hepatic or renal impairment. However, the application of remimazolam in ECT remains unexplored. Prior investigations have associated benzodiazepines with reduced seizure duration in ECT [30, 31], leading to cautious dosing strategies within ECT treatment courses. Park et al. [32] conducted a retrospective comparison of remimazolam versus propofol in ECT (49 patients, 405 ECT sessions), observing comparable motor and EEG seizure durations between propofol and remimazolam. However, evidence remains limited to observational studies and case reports. This randomized controlled trial was designed to evaluate the seizure adequacy and safety of remimazolam in ECT, addressing a critical gap in the pharmacologic optimization of ECT anesthesia.

## Method

### Study design

This study was a single-center, double-blind, prospective, randomized, crossover trial conducted at a tertiary psychiatric hospital in China. It was conducted in accordance with the Declaration of Helsinki, approved by the Ethics Committee of the No.984 Hospital of PLA (approval number: 2025060513; date: June 5, 2025) and prospectively registered at the Chinese Clinical Trial Registry (registration number: ChiCTR2500114332; date: December 10, 2025). All patients provided written informed consent prior to enrollment. The results were reported in accordance with the Consolidated Standards of Reporting Trials (CONSORT) statement [33].

### Participants

Participants were eligible for inclusion if they were aged 18 to 65 years; had a clinical diagnosis of major depressive disorder, bipolar disorder, schizophrenia, or schizoaffective disorder with an established indication for ECT; and provided written informed consent personally or through their legally authorized representatives. For participants lacking decisional capacity, consent was obtained from legally authorized representatives.

Exclusion criteria comprised: (i) known hypersensitivity to remimazolam or propofol; (ii) American Society of Anesthesiologists (ASA) physical status ≥ III; (iii) history of substance use disorder or alcohol dependence within the preceding 12 months.

The first subject signed the informed consent on December 30, 2025, and began the first session of ECT treatment on January 2, 2026. ECT treatment for all enrolled patients was completed in February.

### Randomization and blinding

To ensure balanced allocation between treatment sequences, we employed block randomization with randomly varying block sizes of 4 or 6. Using R software (R Foundation for Statistical Computing, Vienna, Austria), a randomization schedule was generated wherein participants were randomly assigned, within each block, to one of two treatment sequences: sequence A (propofol-remimazolam sequence) or sequence B (remimazolam-propofol sequence).

Following screening and confirmation of eligibility, participants were enrolled sequentially according to the randomization schedule. An independent research nurse, not involved in clinical care or outcome assessment, implemented treatment allocation based on the pre-generated randomization list and prepared study medications. The randomization list was securely stored with restricted access. Allocation concealment was maintained for participants and outcome assessors throughout the trial.

Given the distinct physicochemical properties and administration protocols of remimazolam and propofol, complete blinding of administering anesthesiologists was not feasible. However, we employed a partial double-blind design wherein: (i) participants remained blinded to treatment sequence and specific drug identity; (ii) outcome assessors were blinded to treatment allocation; (iii) the independent research nurse prepared all study medications in identical-appearing syringes labeled with participant identification numbers only, with no involvement in clinical care, data collection, or statistical analysis. We standardized the following blinding procedures: remimazolam was diluted to a concentration of 1 mg/ml to match the injection volume of propofol; all syringes were wrapped in opaque covers immediately after preparation; and the upper extremity with intravenous access was concealed from participants using a screen divider. Both agents were administered as single intravenous bolus doses to achieve the desired depth of anesthesia, with identical injection durations and total administered volumes for both treatment periods to ensure procedural standardization and scientific rigor.

### Intervention administration

**Fig. 1:**
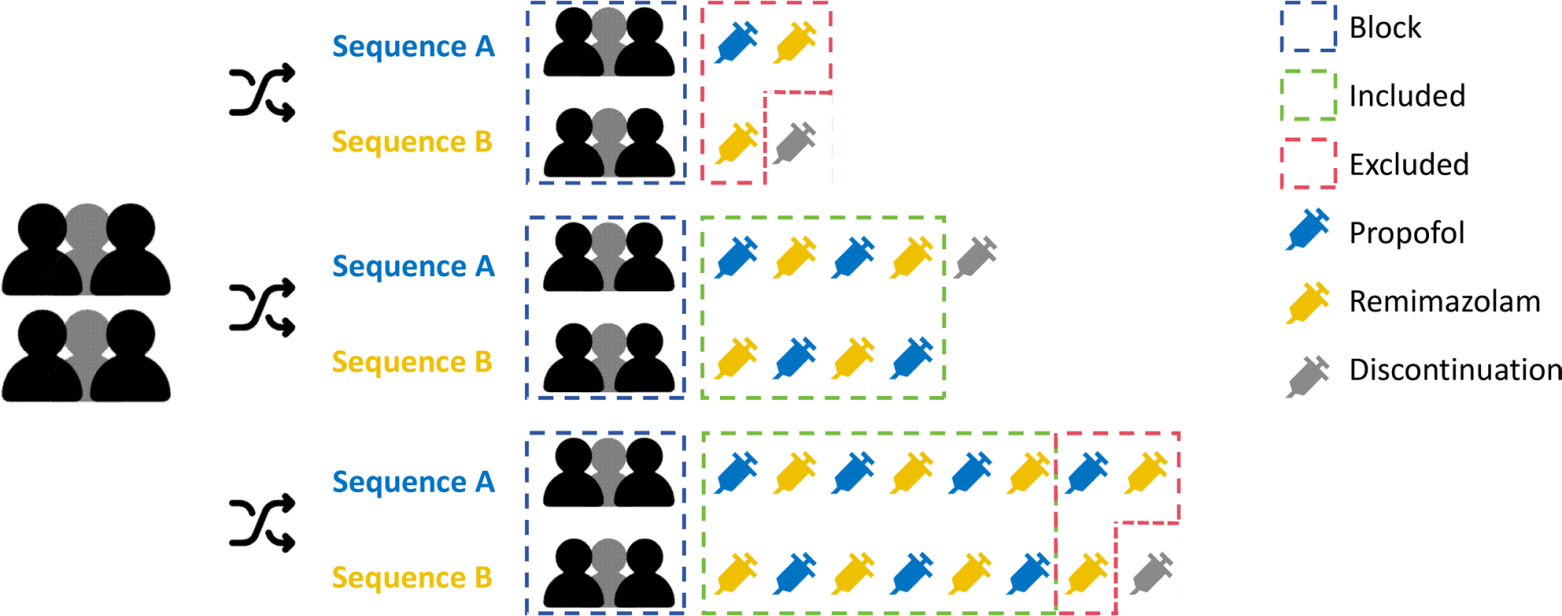
Flow diagram for intervention administration

Participants were enrolled in blocks of 4 or 6 for synchronized scheduling, with equal allocation to the propofol–remimazolam sequence (sequence A) and remimazolam–propofol sequence (sequence B) within each block. All participants within a block commenced ECT sessions concurrently and proceeded through identical alternating schedules, with each pair of consecutive sessions (one propofol, one remimazolam) constituting one treatment period (odd-numbered sessions within each period: sequence-assigned first agent; even-numbered sessions: alternate agent). If any participant discontinued ECT before completing a treatment period, all remaining participants in that block completed their current session but did not proceed to subsequent sessions. This block-level discontinuation preserved allocation balance, prevented confounding between carryover effects and time trends, ensured missing data occurred only at the block level, and guaranteed that every analyzed participant completed both treatment periods, thereby maintaining within-subject comparison integrity. The intervention schedule is illustrated in Fig. 1.

### ECT procedure

All ECT sessions were conducted in the morning. Participants fasted from solids and clear liquids after 22:00 on the evening prior to each treatment to ensure a minimum 8-hour fast, verified by nursing assessment upon arrival. Upon entering the treatment room, participants were positioned supine, and intravenous access was established via an 18- or 20-gauge catheter in one upper extremity. Physiologic monitoring included non-invasive blood pressure, pulse oximetry, and capnography. Seizure quality was assessed using the Thymatron System IV’s integrated digital electroencephalography (EEG), electromyography (EMG) and 3-lead electrocardiography (ECG) module (Somatics, Inc.), which captured: (i) motor seizure duration via frontalis EMG; (ii) ictal EEG duration, amplitude, and post-ictal suppression index (PSI); and (iii) cardiac rhythm throughout the procedure. Raw EEG tracings were reviewed by an investigator blinded to treatment allocation to verify automated measurements.

All participants received total intravenous anesthesia administered by the same attending anesthesiologist. The doses of remimazolam and propofol were selected based on previous studies, with 2.0 mg/kg propofol and 0.2 mg/kg remimazolam used as induction agents for the two sequences [34–37]. Remimazolam (0.2 ml/kg) or propofol (0.2 ml/kg) was administered as an intravenous bolus over 25–30 s. Depth of sedation was assessed using the Modified Observer’s Assessment of Alertness/Sedation (MOAA/S) scale. Upon attainment of MOAA/S score < 3, succinylcholine (1 mg/kg) was administered as an intravenous bolus over 10 s. Following confirmation of loss of spontaneous respiration, controlled positive-pressure ventilation was initiated via an anesthesia facemask with a tidal volume of 6–8 ml/kg and a respiratory rate of 12 breaths per minute. ECT was delivered after allowing approximately 1 min for succinylcholine to achieve full neuromuscular effect, via bilateral frontotemporal placement using a Thymatron System IV device (Somatics, Inc., Lake Bluff, IL, USA) in Low 0.5 mode (constant current 0.9 A, pulse width 0.5 ms). The initial stimulus dose was set at (age − 5)% of the maximal device output, with subsequent dose titration based on seizure adequacy and clinical response by psychiatrists who had received professional training. No restimulation was performed regardless of seizure duration or occurrence, and this standard was applied uniformly to both groups to avoid bias.

Following cessation of the induced seizure, ventilation was transitioned to either continued positive-pressure ventilation or supplemental oxygen at 6 l/min via facemask, based on the return of spontaneous respiration and adequacy of tidal volume. Upon completion of the ECT procedure, participants were transferred to the post-anesthesia care unit (PACU) for a minimum of 30 min. Participants with a modified Aldrete score < 9 at 30 min were retained in the PACU for extended observation until the score reached 9 or higher. Intra-treatment hemodynamic fluctuations and adverse events were managed by the attending anesthesiologist according to standardized protocols.

The treating psychiatrist, blinded to anesthetic allocation, initiated stimulation at the minimum recommended charge dose for the Thymatron System IV device, with subsequent charge adjustments for each session based on seizure characteristics and clinical response from the preceding treatment.

### Primary outcome

The primary outcome was EEG seizure duration, defined as the interval from stimulus delivery to EEG endpoint as automatically detected by the Thymatron System IV device and subsequently verified by manual adjudication of the digital EEG tracing. The endpoint was determined by the cessation of sustained ictal activity, characterized by the return of background rhythm or postictal suppression pattern, with independent confirmation by a psychiatrist blinded to treatment allocation and session number.

### Secondary outcomes

Secondary outcomes comprised seizure adequacy assessments of induced seizure quality, safety evaluations encompassing anesthesia-related parameters, and adverse event monitoring.

Seizure adequacy assessments included: (i) EMG duration; (ii) Average Seizure Energy Index (ASEI); (iii) PSI; and (iv) Maximum Sustained Power (MSP), all automatically recorded by the Thymatron System IV device and verified by manual adjudication. Inadequate seizures were defined as those with an EEG duration of less than 15 s [38, 39].

Anesthesia-related assessments comprised: (i) induction time, defined as the interval from study drug initiation to attainment of MOAA/S score < 3; and (ii) recovery time, defined as the interval from study drug initiation to return of MOAA/S score > 3.

Adverse events were prospectively monitored throughout the peri-procedural period and included: (i) hypertension, defined as maximum mean arterial pressure (MAP) > 150 mmHg during the treatment period; (ii) tachycardia, defined as maximum heart rate > 120 beats per minute; (iii) bradycardia, defined as minimum heart rate < 60 beats per minute post-treatment; (iv) injection pain; (v) dizziness; and (vi) nausea and vomiting.

### Sample size calculation

This randomized crossover trial was designed as a superiority study to demonstrate that remimazolam prolongs EEG seizure duration compared with propofol. We set the significance level at two-sided α = 0.05 and targeted 80% power. Based on an unpublished pilot study (*n* = 20), the within-subject standard deviation of the treatment difference was estimated at 20 s. To detect a clinically meaningful difference (Δ = 20 s, remimazolam superior to propofol), we calculated that 21 participants would be required for a standard two-period crossover design.

To enhance robustness for multi-period comparisons (3 periods per treatment, 6 sessions per participant) and to account for potential period effects and treatment-by-period interactions, we inflated the sample size. With 42 participants completing 6 sessions each, we obtain 126 within-subject treatment pairs, providing 252 ECT sessions. Considering a 20% comprehensive dropout rate (including early discontinuation and incomplete session pairs), we planned to randomize 54 participants, yielding 324 ECT sessions.

### Statistical analysis

All statistical analyses and graphics were performed using R version 4.4.2 (R Foundation for Statistical Computing, Vienna, Austria). Continuous variables were summarized as mean (standard deviation, SD) or median (interquartile range, IQR) depending on distribution; categorical variables were presented as counts (proportions).

Given the crossover design, we employed a generalized linear mixed-effects model (GLMM) framework in which random intercepts were specified at the patient level to account for within-subject correlation. Given the crossover design, we formally tested treatment-by-session interactions for all primary and seizure adequacy outcomes using likelihood ratio tests. For continuous outcomes such as EEG seizure duration, linear mixed-effects models (LMM) were fitted with treatment differences expressed as adjusted mean difference (aMD) and 95% confidence intervals (CI). For binary outcomes, mixed-effects logistic regression models were used, with results reported as adjusted odds ratios (aOR) and 95% CI. Both models included treatment (remimazolam vs. propofol), stimulus charge dose, and treatment period (session number) as fixed effects, with random intercepts for participant. A two-sided *p* value < 0.05 was considered statistically significant. The Holm-Bonferroni method was used to adjust *p* values for multiple comparisons.

## Result

A total of 63 patients with psychiatric disorders scheduled for ECT were screened for eligibility. Three patients were excluded due to age < 18 years (*n* = 3). Sixty participants meeting all inclusion criteria were enrolled and randomized.

One participant withdrew consent and terminated treatment after the first ECT session. Per the prespecified block discontinuation rule, the remaining three participants in the same block were withdrawn, resulting in four participants excluded from analysis.

**Fig. 2:**
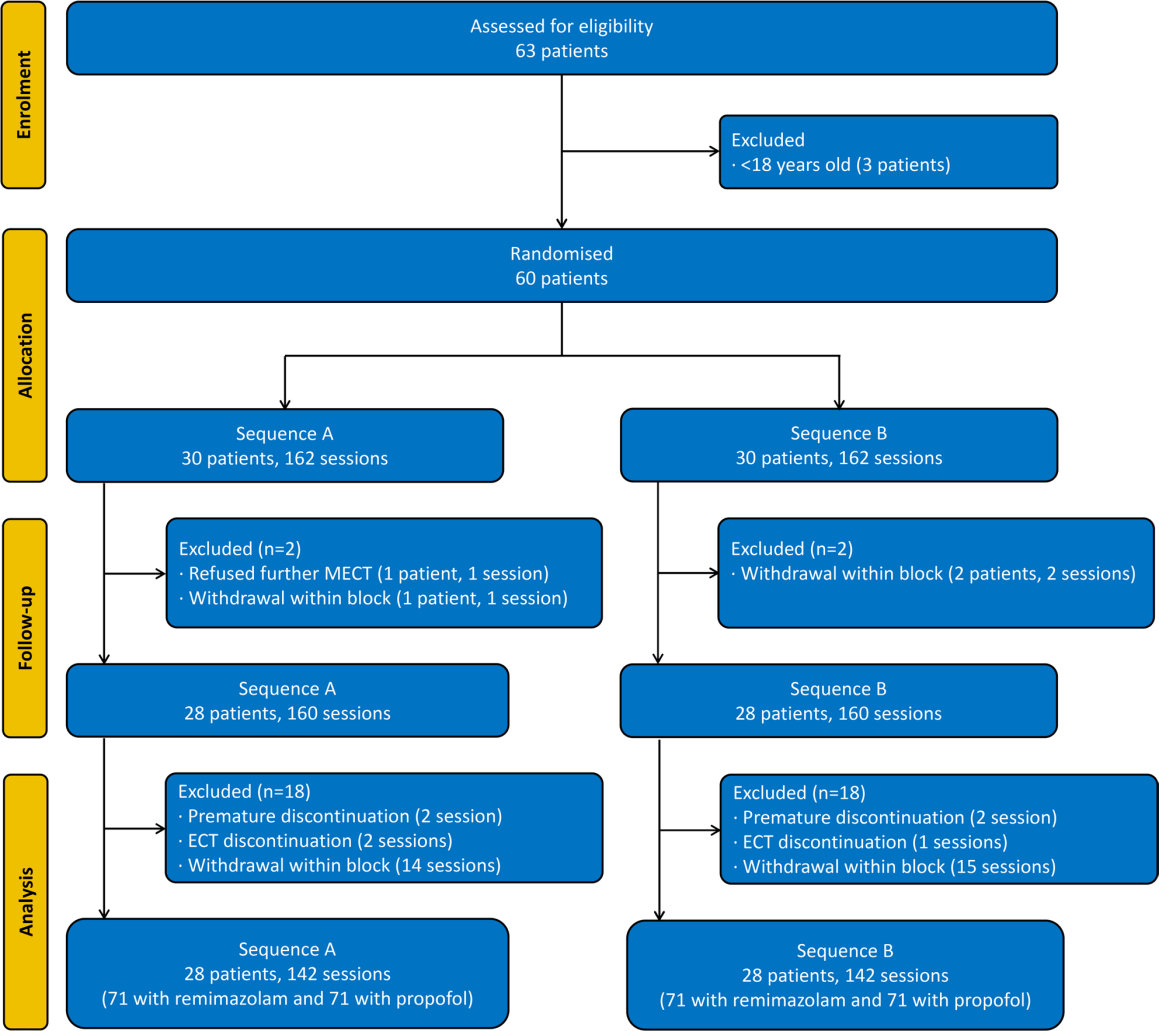
CONSORT flow diagram

During the course of treatment, seven participants prematurely discontinued ECT. Contributed 7 unpaired sessions; additionally, 29 unpaired sessions from the same blocks were excluded per protocol to maintain balanced intervention exposure. This resulted in 56 participants with 284 ECT sessions being included in the final analysis (Fig. 2).

### Baseline characteristics

A total of 56 participants were included in the final analysis. The median age was 26.0 (21.8, 35.8) years, mean body mass index (BMI) was 24.4 ± 4.6 kg/m², and 53.6% (*n* = 30) were classified as ASA physical status I. 10.7% (*n* = 6) had postgraduate education. Across 284 ECT sessions, the median stimulus charge was 150.5 (111.1, 227.0) mC, and median stimulus duration was 6.3 (5.6, 7.0) s. No statistically significant differences were observed between treatment sequences or individual treatments in any baseline or procedural characteristics (all *p* > 0.05) (Tables 1 and 2 & Supplementary Table 1).

**Table 1:** Baseline characteristics of each patient and each session

Baseline characteristics of each patient	Overall	Sequence A	Sequence B	*p* value
Total	56 (100.0)	28 (100.0)	28 (100.0)	
Sex (%)				0.787
Female	24 (42.9)	13 (46.4)	11 (39.3)	
Male	32 (57.1)	15 (53.6)	17 (60.7)	
Age (median (IQR))	26.0 (21.8, 35.8)	26.5 (21.8, 34.2)	25.5 (21.8, 39.2)	0.928
Weight (mean (SD))	70.5 (12.3)	70.8 (12.8)	70.3 (12.0)	0.870
Height (mean (SD))	170.4 (7.5)	170.1 (7.4)	170.8 (7.7)	0.738
BMI (mean (SD))	24.4 (4.6)	24.6 (4.7)	24.2 (4.6)	0.766
ASA (%)				0.180
I	30 (53.6)	18 (64.3)	12 (42.9)	
II	26 (46.4)	10 (35.7)	16 (57.1)	
Diagnose (%)				0.686
SSD	30 (53.6)	16 (57.1)	14 (50.0)	
MD	24 (42.9)	11 (39.3)	13 (46.4)	
AFD	1 (1.8)	1 (3.6)	0 (0.0)	
DD	1 (1.8)	0 (0.0)	1 (3.6)	
Course (median (IQR))	13.0 (2.8, 87.0)	13.0 (2.0, 75.0)	16.5 (3.8, 126.0)	0.838

SSD, Schizophrenia and other primary psychotic disorders; MD, Mood disorders; AFD, Anxiety and fear-related disorders; DD, Dissociative disorders

**Table 2:** Baseline characteristics of each session

Baseline characteristics of each session	Overall	Propofol	Remimazolam	*p* value
Total	284 (100.0)	142 (100.0)	142 (100.0)	
Systolic blood pressure (mean (SD))	79.3 (12.4)	78.6 (11.6)	80.0 (13.1)	0.342
Diastolic blood pressure (mean (SD))	128.2 (17.4)	127.6 (17.0)	128.8 (17.7)	0.553
Mean arterial pressure (mean (SD))	111.9 (15.0)	111.3 (14.5)	112.6 (15.5)	0.477
Heart rate (mean (SD))	76.8 (11.4)	76.2 (11.2)	77.4 (11.6)	0.362
Charge (median (IQR))	150.5 (111.1, 227.0)	151.1 (111.0, 226.9)	150.4 (111.2, 227.0)	0.812
Stimulus duration (median (IQR))	6.3 (5.6, 7.0)	6.3 (5.6, 7.0)	6.3 (5.6, 7.0)	0.358

### EEG duration

**Fig. 3:**
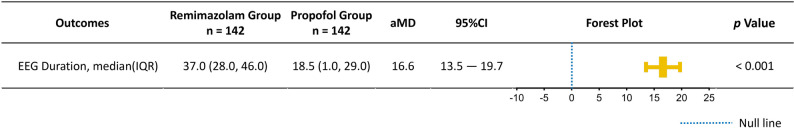
Result of main outcome

**Fig. 4:**
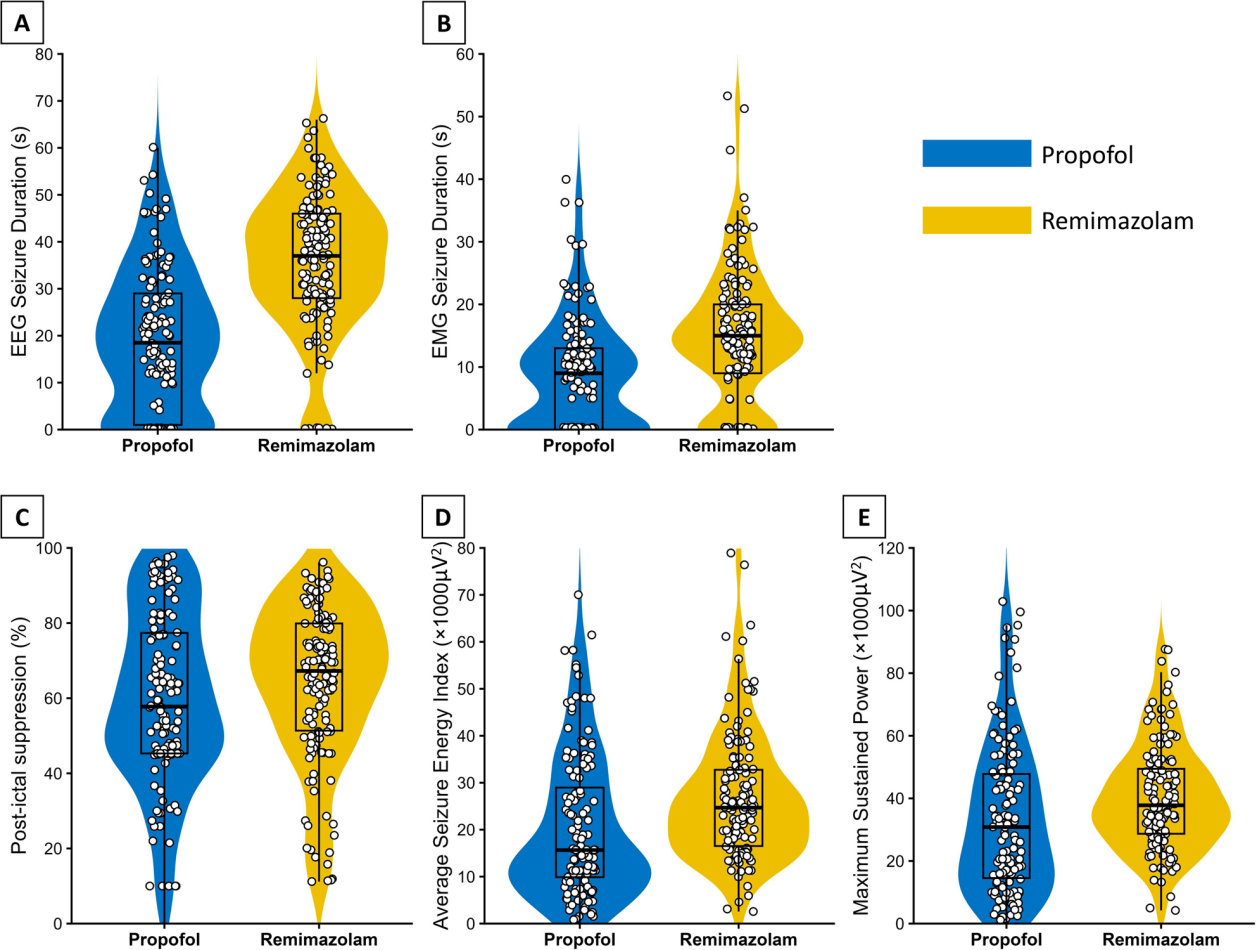
Violin plots showing distribution of seizure adequacy outcomes

In 142 ECT sessions per treatment group, median EEG seizure duration was 37.0 (28.0, 46.0) s with remimazolam versus 18.5 (1.0, 29.0) s with propofol. After adjusting for stimulus charge dose and treatment period as fixed effects in a linear mixed-effects model, remimazolam demonstrated significantly longer EEG seizure duration compared with propofol (aMD: 16.6 s; 95% CI: 13.5 to 19.7 s; *p* < 0.001). The 95% confidence interval excluded zero, confirming the superiority of remimazolam over propofol (Figs. 3 and 4).

### Seizure adequacy outcomes

Remimazolam demonstrated superior seizure quality across multiple metrics compared with propofol. Specifically, EMG seizure duration was significantly prolonged with remimazolam (15.0 [10.0, 22.0] s vs. 9.0 [5.0, 15.0] s; aMD: 6.2 s; 95% CI: 4.1 s to 8.3 s; *p* < 0.001). This enhancement in seizure duration was accompanied by significantly greater seizure intensity, as evidenced by higher ASEI (24,767.4 [19,245.0, 31,580.0] µV^2^ vs. 15,702.7 [11,250.0, 20,340.0] µV^2^; aMD: 9,064.7 µV^2^; 95% CI: 6,520.0 µV^2^ to 11,609.4 µV^2^; *p* < 0.001) and MSP (38,197.7 [32,450.0, 45,680.0] µV^2^ vs. 30,833.7 [25,120.0, 37,560.0] µV^2^; aMD: 7,364.0 µV^2^; 95% CI: 4,890.0 µV^2^ to 9,838.0 µV^2^; *p* < 0.001). Interestingly, despite these improvements in duration and intensity, PSI did not differ significantly between groups (67.3 [55.0, 78.0]% vs. 57.8 [45.0, 70.0]%; aMD: 9.5%; 95% CI: −1.2% to 20.2%; *p* = 0.090), though the point estimate numerically favored remimazolam. Remimazolam was associated with significantly lower odds of inadequate seizures compared to propofol (aOR: 0.1, 95% CI: 0.0 to 0.2, *p* < 0.001). Regarding sessions requiring dose escalation, remimazolam and propofol demonstrated similar escalation probabilities (aOR: 1.1, 95% CI: 0.7 to 1.8, *p* = 0.743). (Table 3; Fig. 4).

**Table 3:** Results for primary and secondary outcomes

Outcomes	Remimazolam *n* = 142	Propofol *n* = 142	aOR/aMD	95%CI	*p* Value
Primary outcome					
EEG Duration, median(IQR)	37.0 (28.0, 46.0)	18.5 (1.0, 29.0)	16.6	13.5 — 19.7	< 0.001
Seizure adequacy outcomes					
EMG Duration, median(IQR)	15.0 (9.0, 20.0)	9.0 (0.0, 13.0)	6.3	4.3 — 8.29	< 0.001^a^
PSI, median(IQR)	67.3 (51.4, 80.0)	57.8 (45.3, 77.4)	4.3	-0.6 — 9.3	0.180^a^
ASEI, median(IQR)	24,767.4 (16,752.2, 33,091.7)	15,702.7 (9,971.9, 31,254.3)	5917.0	2,646.4 — 9,191.5	< 0.001^a^
MSP, median(IQR)	38,197.7 (28,831.1, 50817.4)	30,833.7 (14,541.5, 47791.8)	8619.2	3,996.8 — 13,243.6	< 0.001^a^
Inadequate seizure, n(%)	12 (8.5%)	58 (40.8%)	0.1	0.0 — 0.2	< 0.001^a^
Sessions requiring escalation, n(%)	59 (41.5%)	57 (40.1%)	1.1	0.7 — 1.8	0.743^a^
Safety outcomes					
Induction time, median(IQR)	65.0 (53.0, 81.0)	44.0 (39.0, 50.8)	21.5	18.1 — 25.0	< 0.001
Recovery time, median(IQR)	596.0 (533.5, 650.0)	503.0 (462.0, 555.0)	92.8	74.4 — 111.1	< 0.001
Injection pain, n(%)	1 (0.7%)	19 (13.4%)	0.04	0.01 — 0.32	0.002
Bradycardia, n(%)	2 (1.4%)	2 (1.4%)	1.00	0.14 — 7.34	0.998
Tachycardia, n(%)	140 (98.6%)	98 (69.0%)	39.32	8.79 — 176.02	< 0.001
Hypertension, n(%)	90 (63.4%)	24 (16.9%)	31.93	12.20 — 83.53	< 0.001
Dizziness, n(%)	0 (0.0%)	0 (0.0%)	-	-	-
Nausea and vomiting, n(%)	0 (0.0%)	0 (0.0%)	-	-	-

EEG, electroencephalography; EMG, electromyography; PSI, Post-ictal suppression index; ASEI, average seizure energy index; MSP, Maximum Sustained Power. a. Holm-Bonferroni-adjusted p-values

### Safety outcomes

**Fig. 5:**
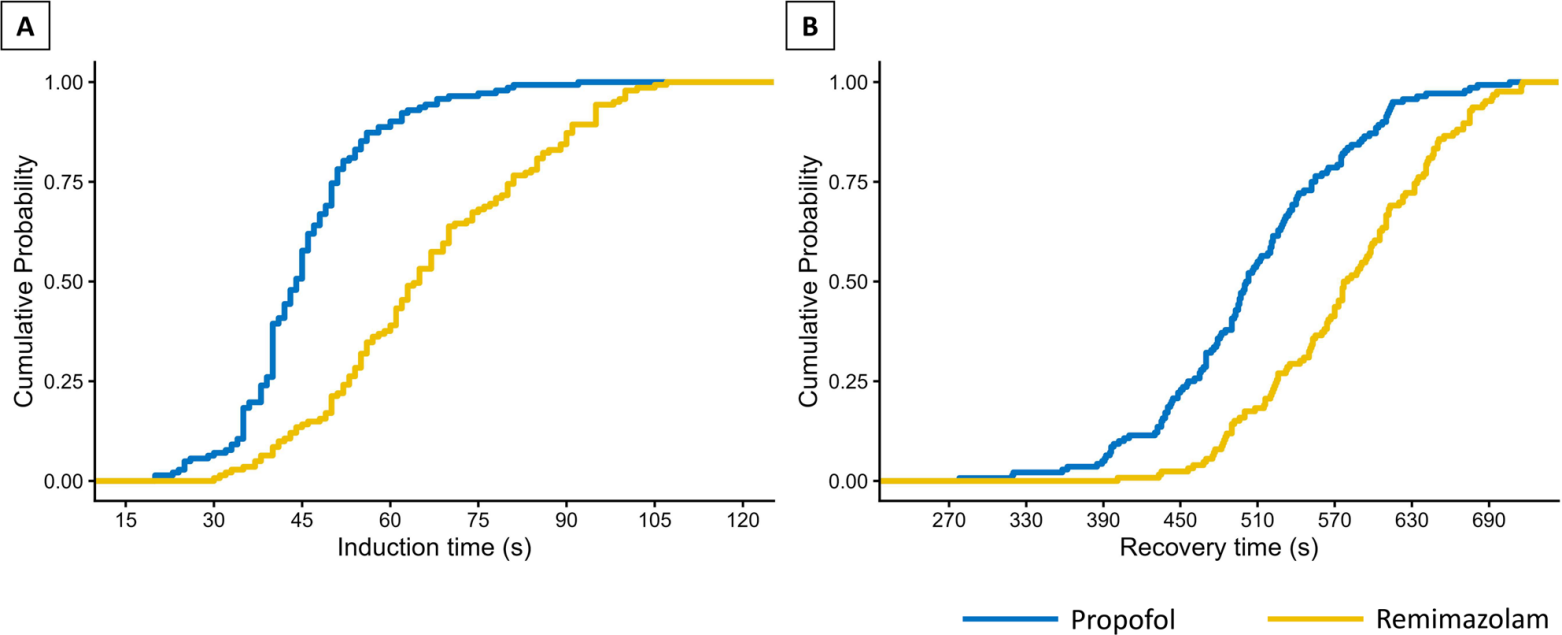
Cumulative curve of induction and recovery times

Remimazolam exhibited significantly longer induction time and recovery time compared with propofol, with induction time of 65.0 (53.0, 81.0) s versus 44.0 (39.0, 50.8) s (aMD: 21.0 s; 95% CI: 17.5 s to 24.5 s; *p* < 0.001) and recovery time of 596.0 (533.5, 650.0) s versus 503.0 (462.0, 555.0) s (aMD: 93.0 s; 95% CI: 76.0 s to 110.0 s; *p* < 0.001) (Fig. 5). However, remimazolam was associated with markedly reduced injection pain, occurring in only 0.7% of sessions compared with 13.4% with propofol (aOR: 0.04; 95% CI: 0.01 to 0.32; *p* < 0.001). Conversely, hemodynamic stimulation was more pronounced with remimazolam, with tachycardia occurring in 98.6% versus 69.0% (aOR: 24.5; 95% CI: 8.2 to 73.4; *p* < 0.001) and hypertension in 63.4% versus 16.9% (aOR: 8.7; 95% CI: 5.1 to 14.9; *p* < 0.001). Notably, no significant between-group differences were observed in the incidence of bradycardia (1.4% vs. 1.4%; *p* = 0.998), dizziness, or nausea and vomiting (Table 3). Given that no participants exhibiting hyperdynamic responses had cardiovascular comorbidities, all hemodynamic events were transient and self-limited without pharmacological intervention: affected participants returned to pre-treatment hemodynamic status within 30 min, and no event required ECT session interruption, protocol modification, or escalation of care.

### Treatment effect

Given the multiple-session crossover design, we formally tested whether the treatment effect varied across sessions using likelihood ratio tests. The treatment-by-session interaction was not statistically significant for EEG duration (χ² = 1.408, *p* = 0.235) and other seizure adequacy outcomes, indicating that the treatment difference remained stable over the course of repeated ECT sessions. Detailed results for all primary and seizure adequacy outcomes are provided in Supplementary Table 2.

### Subgroup analysis

**Fig. 6:**
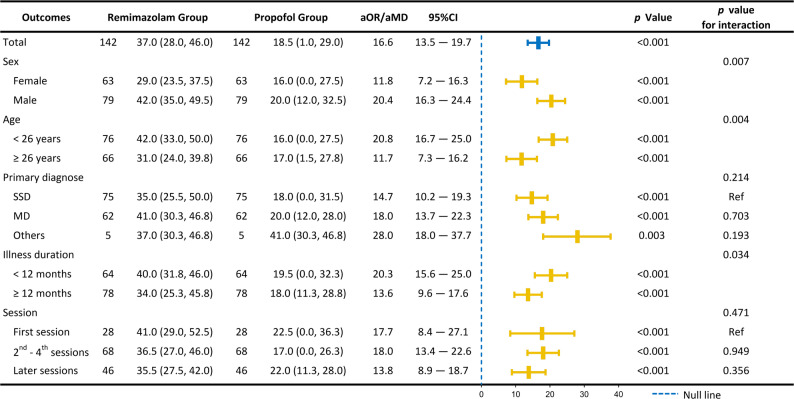
Results of subgroup analysis

We conducted prespecified subgroup analyses to evaluate the consistency of treatment effects across demographic and clinical characteristics, including age, sex, primary diagnosis, duration of illness, and treatment sequence within the ECT course. While statistical heterogeneity was observed across subgroups for sex, age, and illness duration (interaction *p* value < 0.05), the direction of effect consistently favored remimazolam in all subgroups, with the 95% CI for the aMD in EEG seizure duration lying entirely to the right of the null line. These findings indicate that the superior seizure prolongation effect of remimazolam is robust across diverse patient populations and stable throughout the ECT treatment course, with no evidence of carryover effects, supporting the generalizability of the primary results (Fig. 6).

## Discussion

The optimal anesthetic agent for ECT has long remained a subject of debate. The ideal drug should be ultra-short-acting while exerting minimal anticonvulsant effects. Remimazolam, a novel benzodiazepine [27], has been widely adopted for procedural sedation in minor surgeries and outpatient diagnostic procedures [40, 41], attributed to its non-organ-dependent metabolism [42] and favorable hemodynamic profile. Nevertheless, rigorous randomized evaluations of remimazolam in ECT have been lacking.To our knowledge, this is the first randomized trial to evaluate remimazolam in the ECT setting. Our findings demonstrate that, under comparable stimulation parameters, remimazolam at 0.2 mg/kg (compared with propofol at 2.0 mg/kg) elicited significantly prolonged EEG and EMG seizure durations, alongside enhanced ASEI and MSP. This finding contrasts with prior observational data by Park et al. [32] and indicates pharmacodynamic characteristics associated with improved seizure adequacy metrics in this study.

Both remimazolam and propofol exert their anesthetic and sedative effects through potentiation of γ-aminobutyric acid (GABA) ergic neurotransmission, yet they differ markedly in their anticonvulsant properties. Although both agents positively modulate GABA_A_ receptors in the central nervous system [43–45], the structural and functional diversity of GABA_A_ receptor subunits [46] underlies their distinct pharmacological profiles. While benzodiazepines such as remimazolam act at extracellular α/γ sites to increase channel opening frequency, propofol penetrates transmembrane β subunit sites to enhance both opening frequency and conductance [46–49], accounting for its superior anticonvulsant potency. Notably, propofol’s potent anticonvulsant activity has established it as a recommended agent for refractory status epilepticus when benzodiazepines fail [50, 51], whereas remimazolam has no reported application in seizure termination. This relatively attenuated anticonvulsant capacity likely accounts for the remimazolam’s prolonged seizure durations and enhanced seizure adequacy metrics observed with remimazolam in this study. These findings reposition remimazolam’s pharmacologic profile—from a limitation in seizure control [31] to an asset in seizure-dependent therapeutics—potentially offering an alternative for patients with propofol-induced seizure inadequacy.

We observed a higher incidence of post-ECT hyperdynamic states (hypertension and tachycardia) [52] in the remimazolam group (as shown in Table 2). This physiological response represents a complex cascade of intense autonomic nervous system activation and endocrine stress reactions triggered by ECT-induced generalized seizure [53]. Seizure-induced sympathetic activation precipitates acute surges in circulating catecholamines [54], which, through simultaneous overstimulation of α_1_-adrenergic and β1-adrenergic receptors, manifest as severe hypertension and tachycardia. Similar hyperdynamic responses have been observed with etomidate in previous investigations [22]. Previous comparative studies of remimazolam versus propofol have associated propofol with greater hypotension and bradycardia [16, 55]; however, in the context of ECT anesthesia, this circulatory depressant effect paradoxically attenuates post-ictal hyperdynamic responses. While the incidence of tachycardia and hypertension was higher with remimazolam, the absolute hemodynamic perturbations remained within the expected physiological range for ECT and were transient in all cases. Recognizing this transient cardiovascular stress, participants exhibiting hyperdynamic responses received extended PACU monitoring; all affected individuals returned to pre-treatment hemodynamic circulatory status within 30 min without intervention. This pattern is consistent with established ECT literature [14], where sympathetic discharge during the ictal phase commonly produces self-limited hemodynamic fluctuations that rarely necessitate pharmacological management. However, the risk of hemodynamic complications is correspondingly increased in patients with cardiovascular comorbidities, warranting particular vigilance or prophylactic pharmacological intervention in this population.

In this study, both groups initiated treatment at the minimum recommended stimulus dose; however, remimazolam elicited significantly prolonged EEG and EMG seizure durations alongside more pronounced autonomic nervous system activation. These findings suggest that downward titration of the initial stimulus dose may be warranted when using remimazolam for ECT, thereby striking a better balance between seizure adequacy and reduced adverse effects.

In this study, the incidence of injection pain was significantly lower with remimazolam compared with propofol (0.7% vs. 13.4%; *p* < 0.001). This observed rate was markedly lower than previously reported in the literature [56, 57], possibly attributable to the overall more sedated or apathetic state of our study population [58], which may have attenuated subjective reporting or behavioral responses to noxious stimuli.

Remimazolam prolonged both induction and emergence times relative to propofol in this study, with mean differences of 21.5 (95% CI: 18.1 to 25.0) s and 92.8 (95% CI: 74.4 to 111.1) s, respectively. These findings align with most prior investigations [34, 59, 60], as remimazolam did not demonstrate shorter induction or recovery profiles. This phenomenon may relate to the specific characteristics of the psychiatric population undergoing ECT and the unique procedural context. Patients with psychiatric disorders often receive more frequent sedative medications and may develop tolerance to sedative effects [61], ultimately prolonging drug onset time. Furthermore, unlike propofol, remimazolam exhibits a context-sensitive half-time that does not increase with infusion duration [62–64]. However, the advantage of remimazolam’s lack of accumulation typically requires multiple administrations or prolonged continuous infusion to manifest fully [64, 65]; the single-bolus regimen employed in ECT thus limits this benefit, precluding rapid induction and emergence.

An ideal ECT regimen should maximize seizure adequacy and clinical response while minimizing side effects. In light of this, this study represents the first randomized controlled trial employing remimazolam for ECT anesthesia, establishing its efficacy in both anesthetic management and seizure adequacy. Compared with propofol, remimazolam demonstrated better seizure adequacy, longer EEG duration, and higher ASEI and MSP. However, aside from significantly reducing injection pain, remimazolam did not decrease other adverse circulatory events and was associated with a higher incidence of hyperdynamic circulatory status.

This study has several limitations. Firstly, constrained by the intensive treatment schedule of ECT and the crossover trial design, this study evaluated only short-term outcomes, with no long-term follow-up data regarding clinical efficiency, cognitive function or relapse rates, and was unable to characterize dose escalation patterns across different anesthetic agents. Secondly, due to constraints in research funding and patient disease burden, this study relied on previously established dosing regimens for sedative medications [34, 35] rather than conducting anesthetic depth monitoring. Thirdly, Global variations exist in the choice of anesthetic induction agents and their dosages prior to ECT. This study only compared remimazolam with propofol at a single dose of 2.0 mg/kg, which may have amplified the advantage of remimazolam in seizure duration and may affect the applicability of our findings to institutions that use thiopental or methohexital as induction agents and limits the generalizability and external validity of the findings. Future investigations should incorporate larger-scale, parallel-group randomized trials to elucidate the impact of remimazolam anesthesia on initial stimulus dosing requirements and long-term clinical outcomes, including cognitive and memory impairments.

## Conclusion

Remimazolam is a viable anesthetic option for ECT, capable of eliciting prolonged EEG and EMG seizure durations at comparable stimulus intensities. However, clinicians should remain vigilant regarding the increased risk of post-ictal hyperdynamic states.

## Supplementary Information

Below is the link to the electronic supplementary material.

Supplementary Material 1

Supplementary Material 2

## Data Availability

The data that support the findings of this study are available from the corresponding author upon reasonable request. Researchers who wish to access the data are required to submit a request to the corresponding author, detailing the purpose and intended use of the data. The corresponding author will evaluate each request and determine whether to grant access to the data.

## Abbreviations

ECT: Electroconvulsive therapy
aMD: adjusted mean difference
CI: Confidence intervals
ASA: American Society of Anesthesiologists
EMG: Electromyography
ECG: Electrocardiography
MOAA/S: Modified Observer's Assessment of Alertness/Sedation
PACU: Post-anesthesia care unit
MAP: Mean arterial pressure
ASEI: Average seizure energy index
PSI: Postictal suppression index
MSP: Maximum sustained power
SD: Standard deviation
IQR: Interquartile range
GLMM: Generalized linear mixed-effects model
LMM: Linear mixed-effects models
aOR: adjusted odds ratios
GABA: γ-aminobutyric acid
