# Supplementary material for: Seizure adequacy and safety of remimazolam in electroconvulsive therapy for patients with psychiatric disorders: a double-blind randomized crossover trial
Source: BMC Psychiatry. 2026 May 22;26:625. doi: 10.1186/s12888-026-08202-x (PMC13474910; doi:10.1186/s12888-026-08202-x)
Supplement: Supplementary file 1 [file 12888_2026_8202_MOESM1_ESM.docx]

Supplementary Table 1. Baseline characteristics of each patient and each session(continued table).

| **Baseline characteristics of each patient** | Overall | Sequence A | Sequence B | *p* value |
| --- | --- | --- | --- | --- |
| Total | 56 (100.0) | 28 (100.0) | 28 (100.0) |  |
| Marriage (%) |  |  |  | 0.658 |
| Unmarried | 44 (78.6) | 22 (78.6) | 22 (78.6) |  |
| Married | 8 (14.3) | 3 (10.7) | 5 (17.9) |  |
| Divorced | 2 (3.6) | 1 (3.6) | 1 (3.6) |  |
| Widowed | 2 (3.6) | 2 (7.1) | 0 (0.0) |  |
| Smoke (%) |  |  |  | 0.403 |
| No | 36 (64.3) | 20 (71.4) | 16 (57.1) |  |
| Yes | 20 (35.7) | 8 (28.6) | 12 (42.9) |  |
| Drink (%) |  |  |  | 0.705 |
| No | 48 (85.7) | 23 (82.1) | 25 (89.3) |  |
| Yes | 8 (14.3) | 5 (17.9) | 3 (10.7) |  |
| Education (%) |  |  |  | 0.142 |
| Middle or high school | 20 (35.7) | 9 (32.1) | 11 (39.3) |  |
| College | 30 (53.6) | 18 (64.3) | 12 (42.9) |  |
| Postgraduate | 6 (10.7) | 1 (3.6) | 5 (17.9) |  |
| Hypertension (%) |  |  |  | 1.000 |
| No | 54 (96.4) | 27 (96.4) | 27 (96.4) |  |
| Yes | 2 (3.6) | 1 (3.6) | 1 (3.6) |  |
| Diabetes (%) |  |  |  | 0.111 |
| No | 52 (92.9) | 28 (100.0) | 24 (85.7) |  |
| Yes | 4 (7.1) | 0 (0.0) | 4 (14.3) |  |
