# Supplementary material for: Seizure adequacy and safety of remimazolam in electroconvulsive therapy for patients with psychiatric disorders: a double-blind randomized crossover trial
Source: BMC Psychiatry. 2026 May 22;26:625. doi: 10.1186/s12888-026-08202-x (PMC13474910; doi:10.1186/s12888-026-08202-x)
Supplement: Supplementary file 2 [file 12888_2026_8202_MOESM2_ESM.docx]

Supplementary Table 2. Results of treatment-by-sessions interaction test.

| Outcomes | χ² | *p* Value |
| --- | --- | --- |
| Primary outcome |  |  |
| EEG Duration, median(IQR) | 1.408 | 0.235 |
| Seizure adequacy outcomes |  |  |
| EMG Duration, median(IQR) | 2.847 | 0.092 |
| PSI, median(IQR) | 1.638 | 0.201 |
| ASEI, median(IQR) | 1.833 | 0.176 |
| MSP, median(IQR) | 3.814 | 0.051 |
| Inadequate seizure, n(%) | 0.014 | 0.905 |
| Sessions requiring escalation, n(%) | 1.320 | 0.251 |

EEG, electroencephalography; EMG, electromyography; PSI, Post-ictal suppression index; ASEI, average seizure energy index; MSP, Maximum Sustained Power
